# Evolution of radiation-induced temporal lobe injury after intensity-modulated radiation therapy in nasopharyngeal carcinoma: a large cohort retrospective study

**DOI:** 10.1186/s13014-024-02400-1

**Published:** 2024-01-19

**Authors:** Jing Hou, Yun He, Handong Li, Zhaodong Ai, Qiang Lu, Biao Zeng, Chuanmiao Xie, Xiaoping Yu

**Affiliations:** 1 Department of Diagnostic Radiology, Hunan Cancer Hospital and the Affiliated Cancer Hospital of Xiangya School of Medicine, Central South University, Changsha, 410013 Hunan People’s Republic of China; 2 Department of Radiology, Sun Yat-Sen University Cancer Center, Guangzhou, 510060 Guangdong People’s Republic of China; 3 State Key Laboratory of Oncology in South China, Guangdong Key Laboratory of Nasopharyngeal Carcinoma Diagnosis and Therapy, Guangdong Provincial Clinical Research Center for Cancer, Guangzhou, 510060 Guangdong People’s Republic of China; 4 Department of Radiotherapy, Hunan Cancer Hospital and the Affiliated Cancer Hospital of Xiangya School of Medicine, Central South University, Changsha, 410013 Hunan People’s Republic of China

**Keywords:** Temporal lobe injury, Nasopharyngeal carcinoma, Intensity-modulated radiotherapy, Magnetic resonance imaging

## Abstract

**Background:**

Previous studies have demonstrated conflicting findings regarding the initial MRI patterns of radiotherapy-induced temporal lobe injury (RTLI) and the evolution of different RTLI patterns. The aim of this study was to evaluate the initial MRI pattern and evolution of RTLI in patients with nasopharyngeal carcinoma (NPC) by means of a large cohort study.

**Methods:**

Data of patients with RTLI were retrospectively collected from two hospitals between January 2011 and December 2021. The injured lobes were categorized into three patterns based on initial MRI patterns: isolated white matter lesions (WMLs), isolated contrast-enhanced lesions (CELs), and combined WMLs and CELs. The latency period, MRI appearances, and temporal changes in WMLs and CELs were evaluated.

**Results:**

A total of 913 RTLI patients with 1092 injured lobes were included in this study. The numbers of isolated WMLs, isolated CELs, and combined WMLs and CELs identified at the first MRI detection were 7 (0.6%), 172 (15.8%), and 913 (83.6%), respectively. The evolution of bilateral RTLI was different in the same patient, and that of unilateral RTLI combined with WMLs and CELs also may occur asynchronously. The time intervals from the initial MRI detection of isolated WMLs, isolated CELs, combined WMLs and CELs to the last negative MRI scan were 8.6, 8.9 and 11.0 months, respectively. A significant difference was observed in the time intervals between the three patterns (H = 14.287, *P* = 0.001). And the time interval was identified as an independent factor influencing the initial MRI pattern of RTLI after Poisson regression (*P* = 0.002).

**Conclusion:**

Both WMLs and CELs could be the initial and only MRI abnormalities in patients with RTLI. This study is of great significance in accurately diagnosing RTLI early and providing timely treatment options. Additionally, it provides clinical evidence for guidelines on NPC, emphasizing the importance of regular follow-up of NPC patients.

## Background

Radiotherapy remains the mainstay of treatment for nasopharyngeal carcinoma (NPC) due to its complicated anatomic location and unique radiotherapy-sensitivity [1]. Since NPC often represents close proximity and infiltration to skull base, temporal lobes are inevitably included into the target volume despite the use of the more advanced intensity-modulated radiotherapy (IMRT) [2–4]. Radiotherapy-induced temporal lobe injury (RTLI) is one of the most serious complications of NPC [1]. The incidence of RTLI among patients receiving IMRT ranged from 4.6 to 8.5% [5]. RTLI can lead to various neurological symptoms including headache, seizures, focal neurological deficits, and cognitive dysfunction [5, 6]. A clear understanding of the early pattern and evolution of RTLI on MRI is crucial for the early treatment of RTLI and the selection of optimal timing for long-term follow-up of RTLI survivors.

According to the timing of radiotherapy, the pathophysiological response of normal brain tissue to radiation can be divided into three stages: the acute reaction period (days to weeks), early delayed radiation period (1–6 months), and late delayed radiation period (6 months to a few years) [7]. Magnetic resonance imaging (MRI) has been considered a useful and noninvasive method for evaluating RTLI and its evolution [8, 9]. White matter lesions (WMLs) and contrast-enhanced lesions (CELs) are the two main MRI manifestations of late radiation injury and can occur separately or together [10]. However, the natural course of these two common patterns of RTLI is still not fully understood, and there are conflicting findings regarding the initial MRI patterns and the evolution of these two patterns of lesions.

Morphological characteristics of RTLI on MRI have been reported in several studies [7–14]. However, these previous studies had limitations, including small sample sizes and inconsistent results. Wang et al. evaluated RTLI evolution in 124 patients with 192 temporal lobe injuries and concluded that RTLI may be reversible, with WMLs appearing first, followed by CELs [10]. In contrast, Zhou et al. [14] investigated the natural course and evolution of RTLI in 105 patients with NPC after radiotherapy and found that solid-enhanced nodular lesions were the earliest MRI abnormalities of RTLI. Therefore, further research with a larger sample size is required to confirm the initial MRI patterns and evolution of RTLI.

This study aimed to enhance understanding of the initial MRI patterns and evolution of RTLI with a large sample size, which may provide a robust theoretical foundation for early and accurate diagnosis, the selection of appropriate follow-up intervals, and timely treatment options for RTLI.

## Materials and methods

### Study design and patients

This study was approved by the institutional review boards of the two participating hospitals (approval numbers: KYJJ-2021-095 and B2020-417-Y01). The requirement for written informed consent was waived owing to the retrospective nature of the study. Patients were recruited from Hunan Cancer Hospital between January 2014 and December 2021 and Sun Yat-Sen University Cancer Center between January 2011 and December 2021.

The inclusion criteria were as follows: (1) pathologically confirmed diagnosis of NPC, (2) received intensity-modulated radiotherapy (IMRT), and (3) confirmed presence of RTLI through careful review of follow-up MRI images of the head and neck. Patients were excluded if they had (1) other abnormalities in the central nervous system, such as cerebral infarctions, tumors, infections, or NPC invasion into the middle cranial fossa; (2) no regular follow-up MRI data; or (3) other malignancies.

The clinical data of the patients were extracted from a picture archiving and communication system (PACS). Patient data included age, sex, RLTI latency period, hypertension, drinking history, smoking history, TNM stage, T stage, N stage, M stage, and the degree of pathological differentiation. The dosimetric parameters for each temporal lobe were obtained from dose-volume histograms (DVH), including the mean dose (D_mean_), maximum dose (D_max_), and minimum dose (D_min_). Among the 913 patients with NPC included in this study, 10 received only IMRT, and the remaining patients received induction chemotherapy and IMRT or concurrent radiochemotherapy. Additionally, some patients underwent additional antineoplastic therapies such as immunotherapy (n = 46).

### MRI appearances of RTLI

The follow-up criteria for all patients included in this study were in accordance with the NCCN guidelines, namely regular follow-up and MRI scans at 3-month intervals during the first year, 6-month intervals during the second year, and annual follow-up with MRI scans thereafter.

All MRI examinations were reviewed in consensus by two radiologists (radiologists H.J. and L.H.) who had 11 and 7 years of experience, respectively, in head and neck imaging. The RTLI latency period was recorded from the end of IMRT until the time RTLI was first diagnosed using MRI. WMLs were defined as white matter lesions that showed homogeneous high signal intensity on T2-weighted images (T2WI) and low signal intensity on T1-weighted images (T1WI). CELs were defined as lesions with slightly high or high signal intensity on T2WI and enhancement on post-contrast T1WI with or without necrosis. Cysts, which present as well-defined round or oval lesions with very high signal intensities on T2WI and thin or imperceptible walls, were not formally assessed in this study because of their low incidence and frequent occurrence in the late stages of RTLI. Therefore, RTLI was classified into three patterns in this study, according to the initial abnormal MRI patterns: isolated WMLs, isolated CELs, and a combination of WMLs and CELs. The evolution of the RTLI components can be divided into four types: increasing, decreasing or dissolving, static, and fluctuating. In this study, an increase was defined as an overall increase in the size of the injured component, with or without a static phase. Decrease or disappearance was defined as an overall decrease or regression in the injury component, with or without a static phase. Fluctuation refers to an increase followed by a decrease, or a decrease followed by an increase, with or without a static phase.

### MRI protocols

MRI examinations at Hunan Cancer Hospital were conducted using a 1.5-Tesla MRI scanner (Optima MR360, GE Healthcare, Milwaukee, WI, USA) equipped with a head and neck combined coil. The MRI protocols consisted of the following sequences: (1) axial T1-weighted imaging (repetition time (TR)/echo time (TE) 580 ms/7.8 ms, slice thickness 5 mm, number of slices 36, slice space 1 mm, number of excitations (NEX) 2); (2) axial T2-weighted imaging with fat suppression (TR/TE 6289 ms/85 ms, slice thickness 5 mm, number of slices 36, slice space 1 mm, NEX 2); and (3) axial contrast-enhanced T1-weighted spin-echo images (TR/TE:500 ms/8 ms, field of view (FOV) 22 × 22 cm, NEX 2, slice thickness 4 mm, interslice gap 0.8 mm).

MRI examinations at Sun Yat-Sen University Cancer Center were conducted using a 1.5-Tesla MRI scanner (Signa, General Electric, CV/i). The imaging protocols were as follows: (1) axial T1-weighted fast spin-echo images ((TR)/(TE) 420–450/min full, slice thickness 6 mm, number of slices 36, slice space 1 mm, NEX 2); (2) axial T2-weighted fast spin-echo images with fat suppression (TR/TE 3200–3500 ms/85 ms, slice thickness 6 mm, number of slices 36, slice space 1 mm, NEX 2); and (3) axial contrast-enhanced T1-weighted spin-echo images (TR/TE:320–350/min full, FOV 22 × 22 cm, NEX 2, slice thickness 6 mm, interslice gap 1 mm).

### Evolution of individual lesions over time and the relationship between the individual components

To assess the evolution of individual lesions in RTLI, each pattern of lesion was evaluated during follow-up MRI examinations. When brain injury occurred in both temporal lobes simultaneously, the evolution of bilateral temporal lobe brain injury was evaluated during the follow-up. Additionally, the evolution of WMLs and CELs observed simultaneously in the unilateral temporal lobe was assessed during the MRI follow-up.

### Statistical analysis

All statistical analyses were conducted using the SPSS v19.0 software (SPSS Inc., Chicago, IL, USA). Statistical significance was set at *P* value < 0.05 in the two-tailed analyses. The Kruskal–Wallis test, chi-squared test, and Fisher's exact test were used to evaluate the continuous and categorical variables between the groups, whenever appropriate. Poisson regression analysis was used to identify independent predictors of initial RTLI patterns.

## Results

### Patient characteristics

**Fig. 1:**
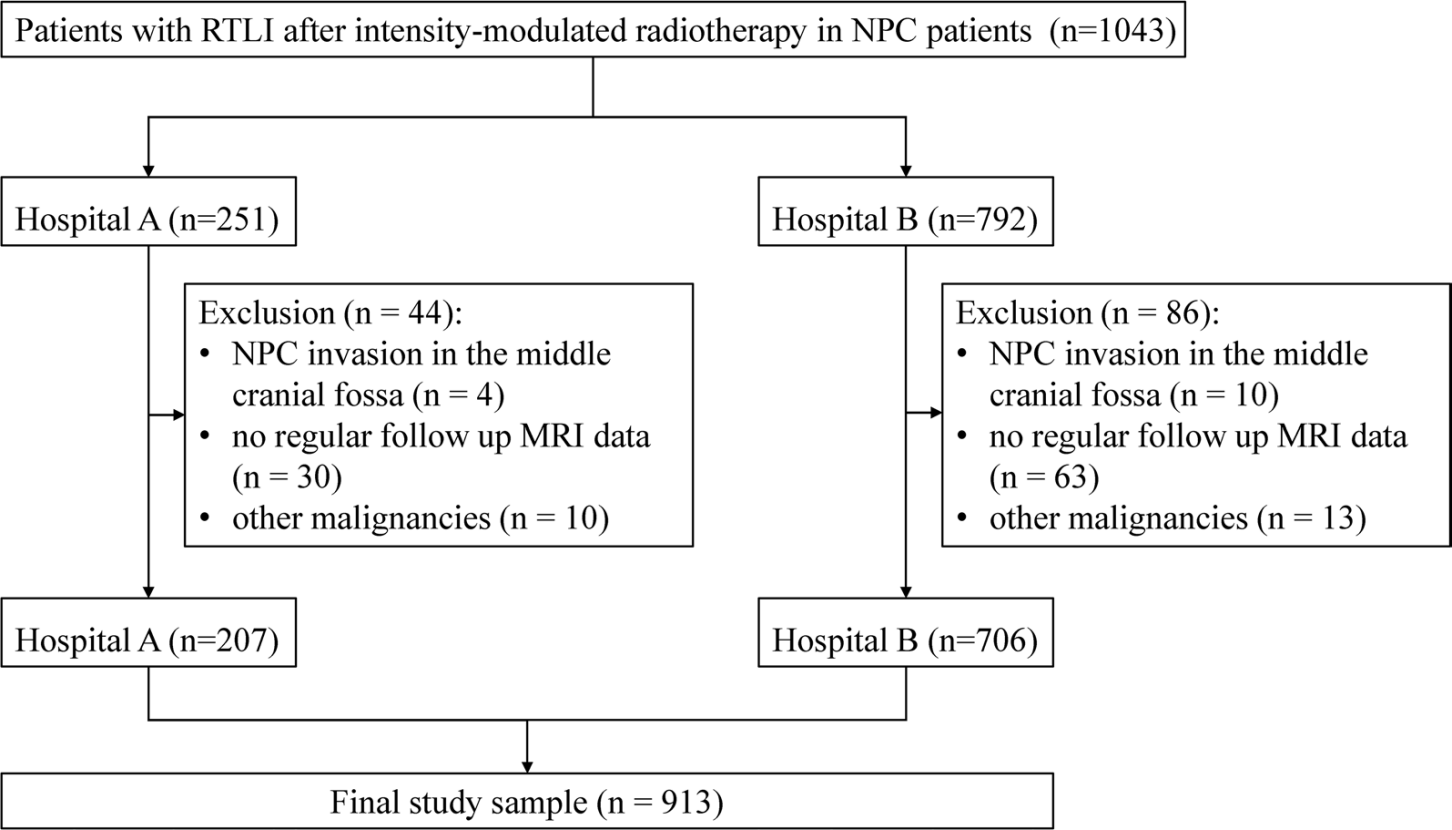
Flow-chart showing the study’s exclusion criteria. *Note*: *NPC* nasopharyngeal carcinoma, *RTLI* radiotherapy-induced temporal lobe injury, *Hospital A*  Hunan Cancer Hospital, *Hospital B* Sun Yat-Sen University Cancer Center

**Fig. 2:**
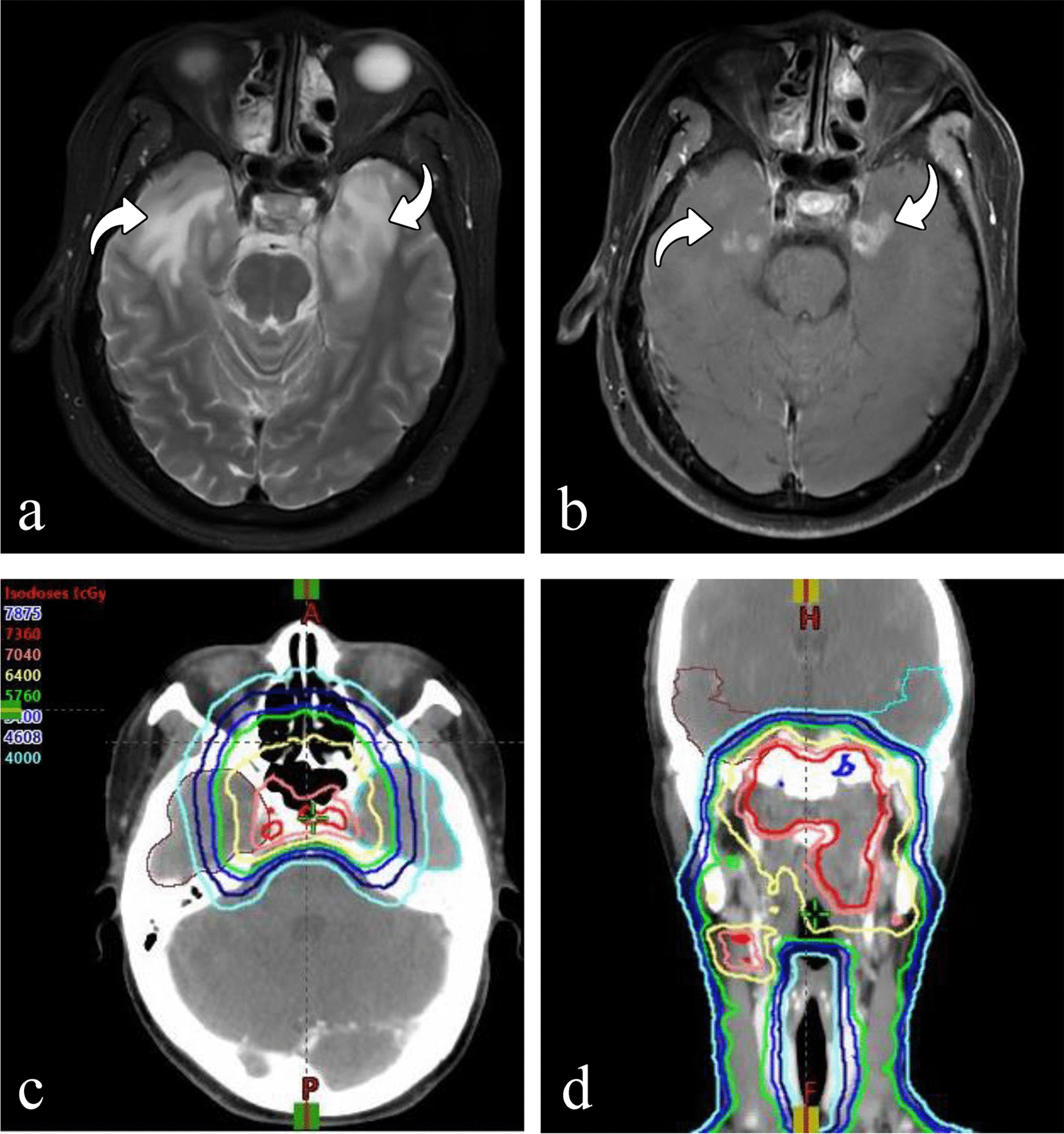
A representative example of white matter lesion in bilateral temporal lobes and dose distribution of the treatment target area treated with three‑dimensional radiotherapy. **a** High intensity in the white matter of bilateral temporal lobes on axial T2-weighted image (curved arrow), **b** contrast-enhanced lesions on axial postcontrast T1-weighted MR image (curved arrow). Dose line distribution is shown in **c** (axial) and **d** (coronal), respectively

Among the 1043 patients with NPC and RTLI screened for this study, 14 were excluded because of NPC invasion into the middle cranial fossa, 93 were excluded because of a lack of follow-up MRI data, and 23 were excluded because of the presence of other malignancies (Fig. 1). Therefore, a total of 913 RTLI patients with 1092 injured lobes were included in this study, with a mean age of 43 ± 12.7 years. Patient characteristics are summarized in Table 1. Among these patients, 734 (80.4%) had unilateral temporal lobe lesions, and 179 (19.6%) had bilateral temporal lobe lesions. Regarding follow-up, 43 patients with RTLI were not followed up with MRI examinations after the initial diagnosis, whereas the remaining 870 underwent at least one MRI follow-up (range, 1–10 examinations), with a total of 2576 follow-up visits. During the follow-up period, 39 new temporal lobe lesions were identified, including three temporal lobes with isolated WMLs, two temporal lobes with isolated CELs, and 34 temporal lobes with CELs combined with WMLs. The D_min_, D_mean_, and D_max_ values for all the included injured temporal lobes were 2.23 ± 1.50, 21.81 ± 6.80, and 74.06 ± 9.16 Gy, respectively. For the individual components of RTLI, the D_min_, D_mean_, and D_max_ values for WMLs were 2.44 ± 1.46, 21.75 ± 6.94, 73.62 ± 5.14 Gy, respectively; for CELs, the values were 2.11 ± 1.10, 21.68 ± 7.95, 73.78 ± 9.53 Gy, respectively; for combination of WMLs and CELs, the values were 2.26 ± 1.57, 21.83 ± 6.56, 74.12 ± 9.12 Gy, respectively. A significant positive correlation was found between the D_mean_ (r = 0.345, *P* = 0.000) and RTLI. Similarly, there was also a significant positive correlation between the D_max_ and RTLI (r = 0.215, *P* = 0.000). Figure 2 displayed a representative example of the radiotherapy plan for NPC and the dose line distribution map of the treatment target area.

**Table 1:** Basic characteristics of patients with NPC and RTLI

Characteristics	No. of patients (%)
Sex	
Male	640 (70.1)
Female	273 (29.9)
Age (years)	
< 50	532 (58.3)
≥ 50	381 (41.7)
Smoking (years)	
Yes	645 (70.6)
No	268 (29.4)
Drinking (years)	
Yes	847 (92.8)
No	66 (8.1)
Hypertension (years)	
Yes	840 (92.0)
No	73 (8.0)
Diabetes (years)	
Yes	894 (97.9)
No	19 (2.1)
Differentiation degree	
Yes	813 (89.0)
No	100 (11.0)
Pathological type	
WHO type 1	0 (0)
WHO type 2	378 (41.4)
WHO type 3	535 (58.6)
TNM stage	
1	2 (0.2)
2	18 (2.0)
3	398 (43.6)
4	495 (54.2)
T stage	
1	12 (1.3)
2	61 (6.7)
3	415 (45.5)
4	425 (46.5)
N stage	
0	51 (5.6)
1	286 (31.3)
2	461 (50.5)
3	115 (12.6)
M stage	
0	907 (99.3)
1	6 (0.7)

*NPC* nasopharyngeal carcinoma, *RTLI* radiotherapy-induced temporal lobe injury

### Initial temporal lobe injury patterns at the first MRI diagnosis of RTLI

**Fig. 3:**
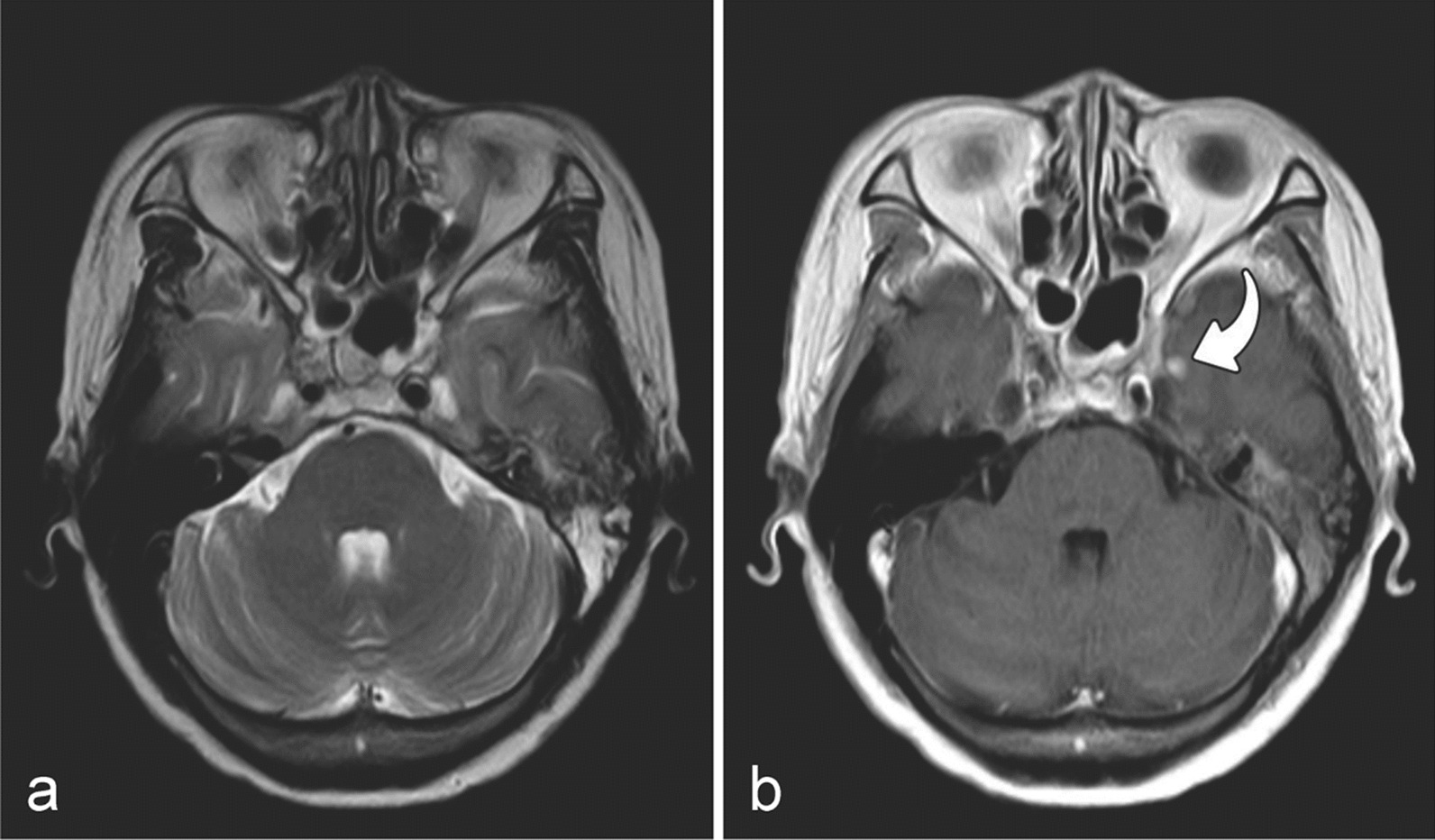
MRIs of a patient with RTLI (46y, M). MRIs of a patient with RTLI (46y, M) with the earliest MRI finding of solid enhanced nodule at 10.87 months after receiving IMRT. Axial T2-weighted image shows slightly high intensity in left temporal lobe (**a**), and a solid enhanced nodule without cystic necrosis is seen in the inferomedial part of the left temporal lobe on the axial post-contrast T1-weighted image (**b**) (curved arrow). *Note*: *MRI* magnetic resonance imaging, *RTLI* radiotherapy-induced temporal lobe injury, *IMRT* intensity-modulated radiotherapy

**Fig. 4:**
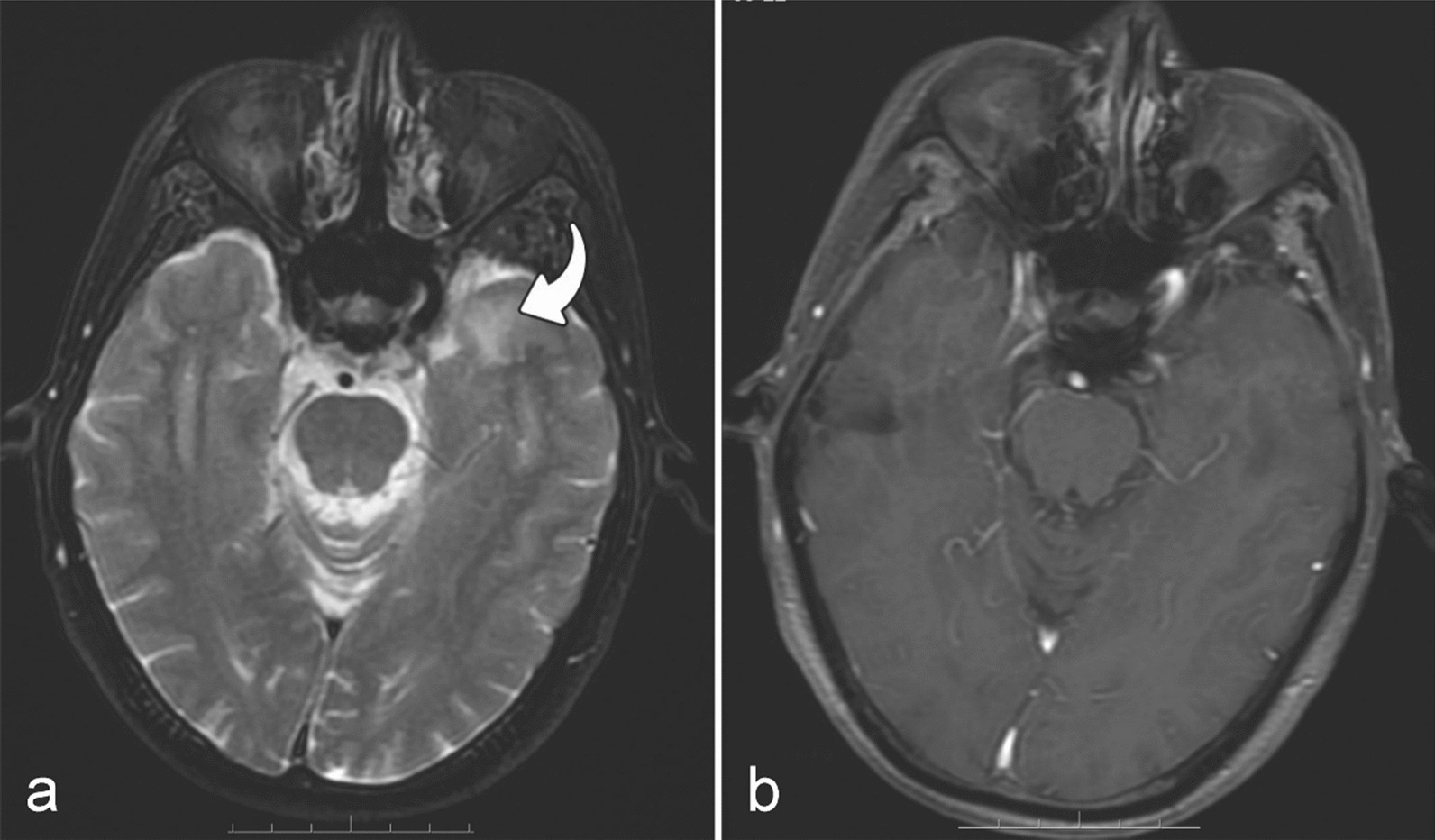
MRIs of a patient with RTLI (65y, F). MRIs of a patient with RTLI (65y, F) with the earliest MRI findings of isolated white matter lesion at 15.33 months after receiving IMRT. Axial T2-weighted image shows high intensity in white matter (curved arrow) of the left temporal lobe (**a**), but no significant enhancement is observed on the axial post-contrast T1-weighted image (**b**). *Note*: *MRI* magnetic resonance imaging, *RTLI* radiotherapy-induced temporal lobe injury, *IMRT* intensity-modulated radiotherapy

Among the 1092 injured lobes, seven (0.6%) had isolated WMLs, 172 (15.8%) had isolated CELs, and 913 (83.6%) had a combination of WMLs and CELs. Initial MRI patterns of the isolated CELs revealed solid enhanced nodules without cystic necrosis (Fig. 3). Initial MRI patterns of the isolated WMLs showed local high T2WI signal in the white matter of the temporal lobe without enhancement (Fig. 4). The median latency time from completion of IMRT to the first MRI detection of RTLI for all injured temporal lobes was 34.1 months (range, 5.7–101.9 months). The mean latency times from the completion of IMRT to the first MRI detection of isolated WMLs, isolated CELs, and combined WMLs and CELs were 28.2, 33.4, and 34.2 months, respectively. Multiple MRI components were detected at longer time intervals after IMRT than individual components (WMLs or CELs); however, the difference in latency time among the three patterns was not statistically significant (H = 2.791, *P* = 0.248).

### Evolution of the individual components of RTLI

The time intervals between the first MRI detection of RTLI and the last negative MRI examination for all the injured lobes were 10.6 months (range, 1.07–79.6 months). Regarding the individual components of RTLI, the time intervals from the first MRI detection of isolated WMLs, isolated CELs, and combined WMLs and CELs to the last negative MRI examination were 8.6, 8.9 and 11.0 months, respectively. Multiple MRI components were detected at a longer time interval after the last negative MRI examination than individual components (WMLs or CELs). The time intervals showed a significant difference among the three patterns (H = 14.287, *P* = 0.001).

**Fig. 5:**
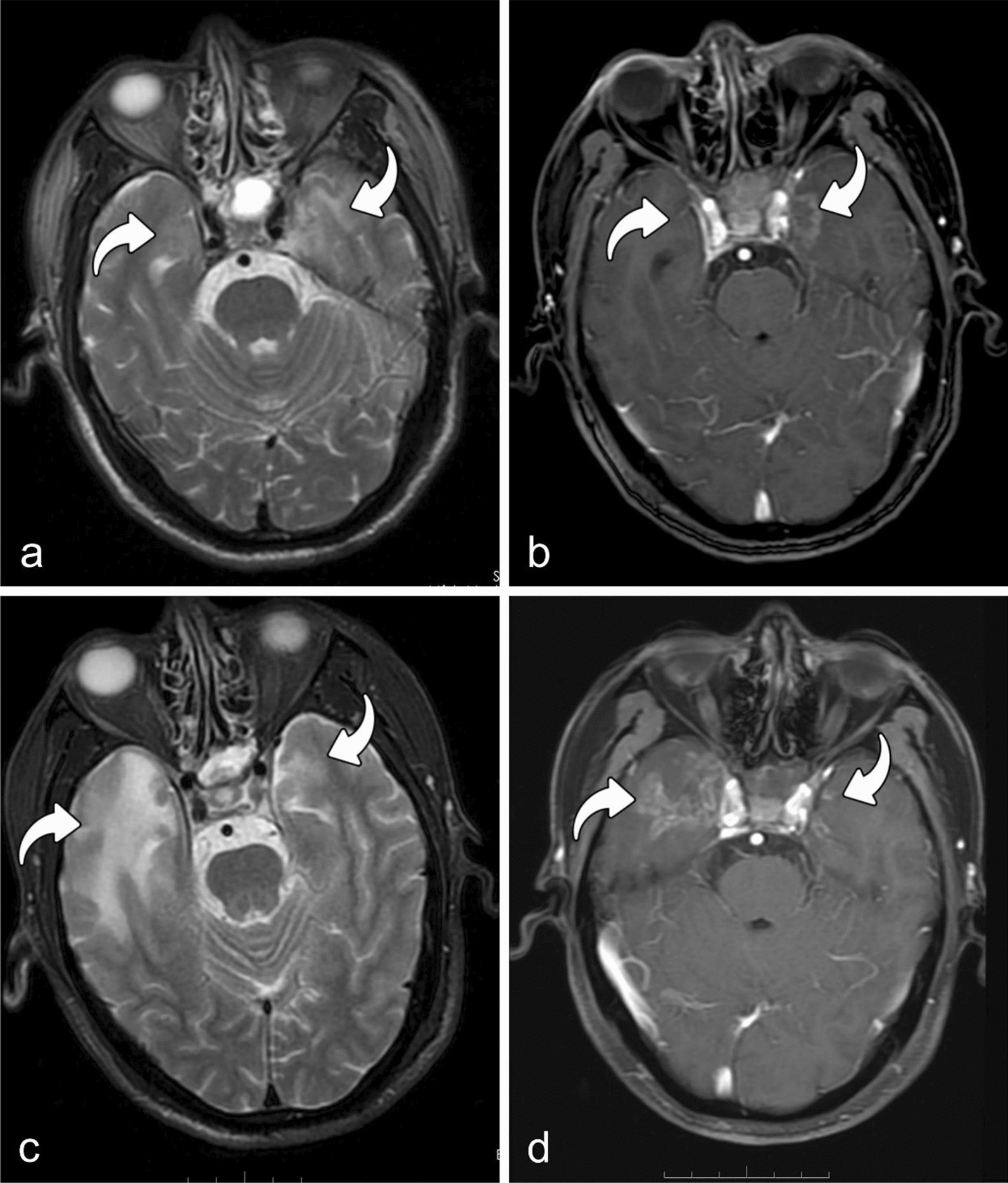
Evolution of bilateral temporal lobe brain injury in one patient (55y, M). Review was conducted at 32.50 months after IMRT. Axial T2-weighted image and post-contrast T1-weighted image show high intensity (**a**) and significant enhancement (**b**) in white matter of the bilateral temporal lobes (curved arrow). After 10.03 months, the white matter lesion and contrast-enhanced lesion increased in the right temporal lobe but regressed in size in left temporal lobe (**c**, **d**) (curved arrow). *Note*: *IMRT* intensity-modulated radiotherapy

**Fig. 6:**
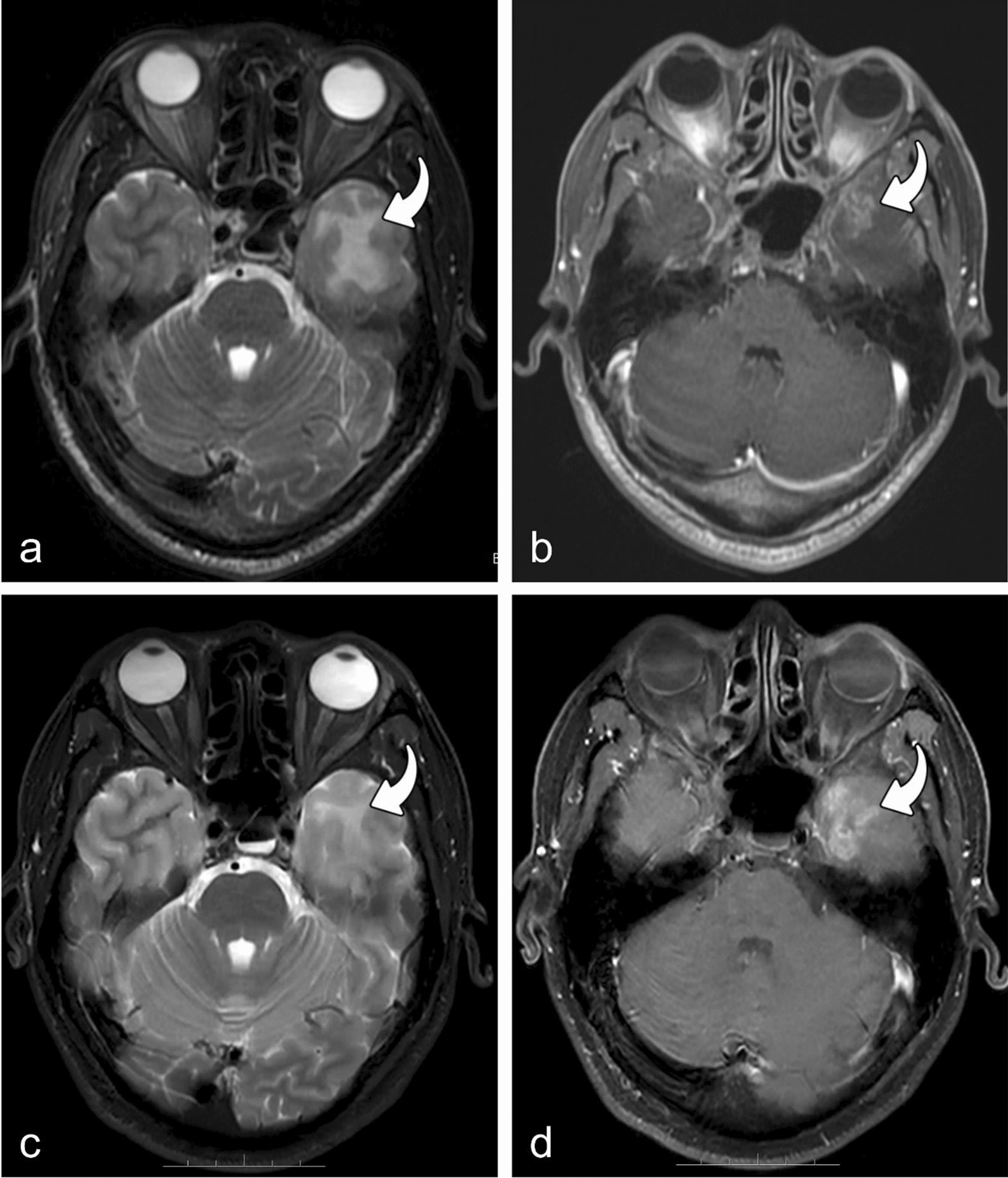
Evolution of individual components of unilateral temporal lobe injury in one patient (43y, M). Review was conducted at 13.87 months after IMRT. Axial T2-weighted image and postcontrast T1-weighted image show hyperintense (**a**) and significant enhancement (**b**) in white matter of left temporal lobe (curved arrow). After 5.73 months, the white matter lesion significantly regressed (**c**), but the contrast-enhanced lesion increased (**d**) (curved arrow). *Note*: *IMRT* intensity-modulated radiotherapy

Furthermore, we observed that the evolution of bilateral temporal lobe brain injury could be different for the same patient (n = 15) (Fig. 5). Additionally, in cases of unilateral temporal lobe injury combined with WML and CEL (n = 119), the evolution of WML and CEL may also be asynchronous (e.g., WML decreasing while CEL increasing) (Fig. 6). The relationship between WMLs and CELs and their changes over time in the unilaterally damaged temporal lobe are presented in Table 2.

**Table 2:** Relationship between WMLs and CELs and their changes over time in unilateral temporal lobe injury

Lesion evolution	CELs increasing, n (%)	CELs decreasing, n (%)	CELs increasing followed by decreasing, n (%)	CELs decreasing followed by increasing, n (%)	CELs stable, n (%)
WMLs increasing	148 (17.2)	8 (0.9)	28 (3.3)	2 (0.2)	5 (0.6)
WMLs decreasing	5 (0.6)	298 (34.6)	17 (2.0)	5 (0.6)	5 (0.6)
WMLs increasing followed by decreasing	9 (1.0)	4 (0.5)	215 (25.0)	1 (0.1)	2 (0.2)
WMLs decreasing followed by increasing	2 (0.2)	1 (0.1)	3 (0.3)	28 (3.3)	0 (0.0)
WMLs stable	6 (0.7)	13 (1.5)	4 (0.5)	1 (0.1)	48 (5.6)

*WMLs* white matter lesions, *CELs* contrast-enhanced lesions

### Relationship between the individual components of RTLI and clinical factors, dosimetric parameters

Significant differences were observed among the three patterns of RTLI regarding T stage (*P* = 0.028), TNM stage (*P* = 0.002), and degree of differentiation (*P* = 0.031) using the chi-squared test or Fisher’s exact test. Additionally, the Kruskal–Wallis test revealed significant differences in age and time interval between the first RTLI detection and the previous negative MRI examination among the three RTLI patterns. Further analysis using Poisson regression identified age (*P* = 0.005) and the time interval between the first RTLI detection and the previous negative MRI examination (*P* = 0.002) as independent factors influencing the three patterns of RTLI (Table 3).

**Table 3:** Poisson regression of the clinical factors for the earliest MRI patterns of RTLI

Variables	b value	Wald X^2^	*P*	RR	95%CI
Age	− 0.001	7.86	0.005	0.999	[0.999, 1.000]
Time interval^a^	0.002	10.0	0.002	1.002	[0.999, 1.002]

*MRI* magnetic resonance imaging, *RTLI* radiotherapy-induced temporal lobe injury

^a^Time interval refers to the period between the first MRI detection of RTLI and the last negative MRI examination

## Discussion

To the best of our knowledge, this study is the largest cohort investigation of the evolution of RTLI in NPC. We found that both WMLs and CELs may manifest as the earliest and sole MRI abnormalities in RTLI. Notably, the evolution of bilateral temporal lobe brain injuries could be different within the same patient, and the evolution of unilateral temporal lobe injury combined with WMLs and CELs may not occur synchronously. In addition, our results suggest that the time interval between the initial detection of RTLI and the last negative MRI examination is an independent predictor of the earliest MRI pattern of RTLI.

Regarding the earliest MRI pattern in RTLI, the results of this study do not completely align with those of previous studies [10, 11]. Wang et al. [10] and Chan et al. [11] reported that WMLs were the earliest abnormal MRI findings of RTLI, with CELs occurring after or concurrently with WMLs. Animal studies have demonstrated that WMLs are the earliest forms of radiation injury [6, 15]. However, Zhou et al. [14] reported that solid-enhanced nodular lesions were the earliest and only initial abnormalities of RTLI. A study by Chan et al. [11] evaluating the morphological characteristics of RTLI on MRI reported a result similar to that of Zhou et al. [14] in which two temporal lobes with blood–brain barrier disruption showed no evidence of white matter lesions. In our study, we found that both WMLs and CELs were the earliest and only MRI patterns associated with RTLI, with isolated CELs having a much higher incidence than isolated WMLs (15.8% vs. 0.6%). The discordance between these studies can be attributed to several reasons. Firstly, the mechanism of radiation-induced brain injury remains unclear and may involve microvascular injury [16], neuronal and neural stem cell (NSC) injury [17–19], glial cell injury [20], inflammation and free radical production [21, 22]. These changes can lead to acute disruption of the blood–brain barrier, increased permeability, and edema [23–25]. Different pathological changes in RTLI may result in different initial manifestations on MRI. Secondly, the follow-up intervals were significantly different among the different studies. In our study, the median time interval between the last previous negative MRI examination and subsequent detection of RTLI on MRI was 10.6 months, which was very close to the 10.5 months reported by Zhou et al. [14], but much shorter than the 20.5 months reported by Wang et al. [10]. Additionally, the time intervals from the first MRI detection of isolated WMLs, isolated CELs, and combined WMLs and CELs to the last previous negative MRI examination in our study were 8.6, 8.9, and 11.0 months, respectively, similar to Zhou et al.’s [14] results demonstrating a shorter time interval between the first MRI detection of CELs and the last negative MRI compared to that in those with multiple MRI components at the first detection of RTLI (5.5 vs 10.5 months, respectively). Moreover, the time interval between the first detection of RTLI and the last previous negative MRI examination was identified as an independent factor influencing the earliest MRI patterns of RTLI based on the results of the Kruskal–Wallis test and Poisson regression. In theory, shorter intervals between MRI examinations may provide more precise information about the natural history of RTLI. However, in practice, some patients did not adhere to regular follow-up, which resulted in prolonged intervals and delayed detection of lesions. Therefore, regular follow-up and MRI examinations, as recommended by guidelines, are crucial for early detection of RTLI and understanding the early MRI pattern of RTLI.

In this study, we observed that the evolution of bilateral temporal lobe injuries varied within the same patient, which supports the evolutionary pattern previously reported by Wang et al. [10]. Additionally, contrary to the commonly accepted principle, we observed that the evolution of WML and CEL may be not synchronized in cases of unilateral temporal lobe injury combined with WML and CEL. Specifically, an increase in CELs may not be accompanied by a corresponding increase in WMLs, and WMLs may decrease or remain static in some cases. Previous studies have reported a positive correlation between edema volume and the incidence of enhancement and necrosis [3, 10], however, our study is the first to demonstrate that the evolution of WMLs and CELs may not occur synchronously. The exact reason for this phenomenon is not fully understood.

The median time interval between the completion of radiotherapy and the first MRI detection of RTLI showed significant variation across studies. In our study, the median interval time was 34.1 months (range, 5.7–101.9 months), which aligns with the findings of Zeng et al. [4] who reported a median interval of 33 months. However, our results indicate a slightly shorter interval (34.1 months) than the 36 and 37 months reported by Wang et al. [10] and Zhou et al. [14], respectively, and a much shorter interval than the 55.9 and 44.5 months reported by Norris et al. [26] and Mao et al. [27], respectively. According to the NCCN guidelines, regular follow-up and MRI examinations should be performed at 3-month intervals during the first year, 6-month intervals during the second year, and yearly intervals thereafter for patients with NPC [28]. Nevertheless, a small number of patients in our study did not undergo routine follow-up MRI. Patients with RTLI typically do not exhibit symptoms in the early stages, and the majority of temporal lobe injuries are incidentally discovered during later reviews. As a result, initial MRI detection of RTLI can occur at any stage of disease progression. This limitation hinders the accurate assessment the exact timing of the initial onset of RTLI and its subsequent evolution.

In this study, we investigated the relationship between the dosimetric parameters and the initial RTLI patterns. However, our findings revealed no significant differences in the dosimetric parameters, including D_min_, D_max_, and D_mean_, among the three RTLI patterns. Radiation dose is considered a direct causal factor for RTLI [6, 29–32]. A previous study confirmed significant differences in dosimetric parameters between RTLI-positive and RTLI-negative lobes in patients with NPC [33]. Additionally, a study using a rat model concluded that the time interval from radiotherapy completion to the onset of RTLI was dose-dependent; however, once the initial onset occurred, the rate of injury progression and total volume generated remained constant across different doses [34]. Therefore, we speculate that the radiation dose impacts the occurrence and timing of radiation-induced brain injury but does not influence the pattern of initial MRI manifestations of RTLI.

This study had several limitations. Firstly, due to the retrospective nature of the study, routine MRI follow-up was not conducted in all patients, which prevented the continuous observation of the entire dynamic process of RTLI evolution. Secondly, less common RTLI patterns, such as gray matter lesions, hemosiderin deposits, and microbleeding foci, were not included in the current study. The main objective of this study was to evaluate the two common MRI manifestations of RTLI, WMLs and CELs, and their temporal changes. Including an analysis of all types of radiation-induced brain injuries would divert the focus of this study. Additionally, the above-mentioned less common patterns are not the primary imaging manifestations of RTLI and typically appear in the later stages.

In conclusion, we found that both WMLs and CELs could be the earliest and only MRI abnormalities associated with RTLI. In addition, to the best of our knowledge, our study indicated for the first time that the evolution of WMLs and CELs may not be synchronized in cases of unilateral temporal lobe injury combined with WMLs and CELs. Furthermore, the time interval between the initial detection of RTLI and the last MRI examination was identified as an independent factor influencing the earliest MRI patterns of RTLI. Regular follow-up intervals can provide more accurate information about the true nature of RTLI. These results are of great significance for the accurate diagnosis of RTLI and timely treatment options. Moreover, the mechanisms underlying the different initial MRI patterns of RTLI are also distinct. Studying the mechanisms behind the initial onset of RTLI may provide possibilities for early mechanism-based interventions. From this perspective, this study provides valuable insights for endeavors aimed at unraveling the mechanisms underlying the occurrence of RTLI.

## Data Availability

The datasets generated or analyzed during the study are available from the corresponding author on reasonable request.

## Abbreviations

CELs: Contrast-enhanced lesions
D_max_: Maximum dose
D_min_: Minimum dose
D_mean_: Mean dose
IMRT: Intensity modulated radiotherapy
MRI: Magnetic resonance imaging
NPC: Nasopharyngeal carcinoma
RTLI: Radiotherapy-induced temporal lobe injury
WMLs: White matter lesions
