# Correction: Evolution of radiation-induced temporal lobe injury after intensity-modulated radiation therapy in nasopharyngeal carcinoma: a large cohort retrospective study

**DOI:** 10.1186/s13014-024-02423-8

**Published:** 2024-03-27

**Authors:** Jing Hou, Yun He, Handong Li, Zhaodong Ai, Qiang Lu, Biao Zeng, Chuanmiao Xie, Xiaoping Yu

**Affiliations:** 1 Department of Diagnostic Radiology, Hunan Cancer Hospital and the Affiliated Cancer Hospital of Xiangya School of Medicine, Central South University, 410013 Changsha, Hunan People’s Republic of China; 2 Department of Radiology, Sun Yat-Sen University Cancer Center, 510060 Guangzhou, Guangdong People’s Republic of China; 3 State Key Laboratory of Oncology in South China, Guangdong Key Laboratory of Nasopharyngeal Carcinoma Diagnosis and Therapy, Guangdong Provincial Clinical Research Center for Cancer, 510060 Guangzhou, Guangdong People’s Republic of China; 4 Department of Radiotherapy, Hunan Cancer Hospital and the Affiliated Cancer Hospital of Xiangya School of Medicine, Central South University, 410013 Changsha, Hunan People’s Republic of China

**Correction: Radiation Oncology (2024) 19:9**

10.1186/s13014-024-02400-1

After publication of this article, the authors reported that in the caption to Fig. 5, the phrase “After 10.03 months, the white matter lesion and contrast-enhanced lesion regressed in the right temporal lobe but increased in size in the left temporal lobe (**c, d**)” should have read “After 10.03 months, the white matter lesion and contrast-enhanced lesion increased in the right temporal lobe but regressed in size in the left temporal lobe (**c, d**)”.

The original article has been corrected.

